# Disruption of the Cdc42/Par6/aPKC or Dlg/Scrib/Lgl Polarity Complex Promotes Epithelial Proliferation via Overlapping Mechanisms

**DOI:** 10.1371/journal.pone.0159881

**Published:** 2016-07-25

**Authors:** Gregory V. Schimizzi, Meghan T. Maher, Andrew J. Loza, Gregory D. Longmore

**Affiliations:** 1 ICCE Institute, Washington University School of Medicine, St. Louis, Missouri, United States of America; 2 Department of Cell Biology and Physiology, Washington University School of Medicine, St. Louis, Missouri, United States of America; 3 Department of Medicine, Washington University School of Medicine, St. Louis, Missouri, United States of America; 4 Department of Computational and Molecular Biophysics, Washington University School of Medicine, St. Louis, Missouri, United States of America; University of Dayton, UNITED STATES

## Abstract

The establishment and maintenance of apical-basal polarity is a defining characteristic and essential feature of functioning epithelia. Apical-basal polarity (ABP) proteins are also tumor suppressors that are targeted for disruption by oncogenic viruses and are commonly mutated in human carcinomas. Disruption of these ABP proteins is an early event in cancer development that results in increased proliferation and epithelial disorganization through means not fully characterized. Using the proliferating *Drosophila melanogaster* wing disc epithelium, we demonstrate that disruption of the junctional vs. basal polarity complexes results in increased epithelial proliferation via distinct downstream signaling pathways. Disruption of the basal polarity complex results in JNK-dependent proliferation, while disruption of the junctional complex primarily results in p38-dependent proliferation. Surprisingly, the Rho-Rok-Myosin contractility apparatus appears to play opposite roles in the regulation of the proliferative phenotype based on which polarity complex is disrupted. In contrast, non-autonomous Tumor Necrosis Factor (TNF) signaling appears to suppress the proliferation that results from apical-basal polarity disruption, regardless of which complex is disrupted. Finally we demonstrate that disruption of the junctional polarity complex activates JNK via the Rho-Rok-Myosin contractility apparatus independent of the cortical actin regulator, Moesin.

## Introduction

A defining feature of epithelia is the presence of apical-basal polarity (ABP), or the asymmetric distribution of lipids, proteins, and mRNAs within the cell. This asymmetric distribution of subcellular components allows for specialized regional function. ABP is also required for the proper establishment and maintenance of intercellular junctions, normal mitoses, and vesicular trafficking. ABP influences cell proliferation and cell migration, and mutations in components of the various ABP complexes can occur early in carcinoma development and contribute to increased proliferation, invasion and metastasis [1,2]. Defects in ABP regulation also cause multilayering and central lumen loss in epithelial tissues, both of which are commonly seen in early stages of cancer [3,4].

Genetic disruption of the basolateral Scribble (Scrib)/Discs Large (Dlg)/Lethal Giant Larvae (Lgl)-complex, or the junctional Cdc42/Par6/Par3/Atypical PKC (aPKC) complex, but not the apical Crumbs/Pars/Pals complex in *D*. *melanogaster* imaginal disc epithelia contribute to dysplastic overgrowth [1,5], but the precise mechanisms of how this occurs and whether through unique or shared pathways remain unclear.

Disruption of the basolateral polarity complex in epithelial cells results in apoptosis. These dying cells can then secrete proliferative signals to neighboring epithelial cells, which proliferate to replace lost cells. If polarity disruption is combined with expression of the baculovirus caspase inhibitor P35 [6], apoptosis execution is blocked and these “undead” cells continue to secrete proliferative signals, leading to tissue overgrowth [7–9]. These proliferative phenotypes involve activation of c-Jun N-terminal Kinase (JNK in mammals; Basket (Bsk) in *D*. *melanogaster*) [1,5,10]. Disruption of ABP also causes an upregulation of Wingless (Wg, *D*. *melanogaster* Wnt) and Decapentaplegic (Dpp, *D*. *melanogaster* TGF-β) signaling in neighboring wild type cells, that contributes to the proliferative phenotypes [7].

While some components required for proliferation downstream of basolateral and junctional ABP disruption are known, a full understanding of how this process occurs is lacking. Moreover, whether disruption of the different ABP complexes lead to proliferative phenotypes through shared or unique pathways is not known. Here we examine signals that result from, or are associated with, disruption of the junctional versus basolateral ABP complex in the developing *Drosophila melanogaster* imaginal wing disc, a proliferating epithelium. Our results demonstrate that interruption of junctional or basolateral ABP regulation leads to distinct downstream signaling events.

## Materials and Methods

### Fly Stocks and Genetics

All crosses and staging were performed at 25°C unless otherwise noted. *w1118* was used as wild-type. Stocks are described in FlyBase (http://flybase.org/). pucE69, UAS-P35 (B#5073), UAS-Scrib-RNAi (B#29552), UAS-Bsk-RNAi (B#32977), UAS-Moesin-myc (B#8631), UAS-Moesin-RNAi (B#8629), UAS-Mekk1-RNAi (B#28587), UAS-Ask1-RNAi (B#32464), UAS-Hep-RNAi (B#28710), UAS-Mkk4-RNAi (B#35140), UAS-p38b-RNAi (B#35252), UAS-p38a-RNAi (B#34744), bsk1 (B#3088), UAS-Baz-RNAi (B#39072), UAS-aPKC-RNAi (B#25946) and “Dlg-RNAi #2” (B#35286) were provided by the Bloomington Drosophila Stock Center (BDSC); Zip1 by T. Wolff (NIH Janelia Research Campus); patched-GAL4 by R. Cagan (Mount Sinai School of Medicine, New York); UAS-Cdc42- RNAi and UAS-Rho1-RNAi were described previously [11,12]; “UAS-Dlg-RNAi #1”(V#41134), UAS-Rok-RNAi (V#3793), and UAS-Wnd-RNAi (V#103410) were provided by the Vienna Drosophila RNAi Center (VDRC); UAS-Slpr-RNAi, UAS-Wengen-RNAi, and UAS-Tak1-RNAi were provided by R. Fehon (University of Chicago); UAS-Egr was provided by M. Vidal (Beatson Institute, Glasgow, UK). MARCM clones were generated by heat shock at 37C for 1h and dissected 40 to 48 hours after clone induction (ACI).

### Immunofluorescence and Image processing

Wandering 3^rd^ instar larval wing discs were dissected in phosphate-buffered saline (PBS), fixed in 4% paraformaldehyde diluted in PBS for 40 min, washed once for five minutes in PBX (PBS with 0.1% Triton X-100), twice for 20min in PAXD (PBS with 1% bovine serum albumin, 0.3% Triton X-100, and 0.3% deoxycholate), and once for 20min in PAXDG (PAXD with 5% normal goat serum), all on ice. Tissues were incubated overnight in primary antibody diluted in PAXDG at 4°C and washed three times in PBX at room temperature. After four or more hours incubated in secondary antibody diluted in PAXDG at 4°C, they were washed twice in PBX, and washed once in PBS, all at room temperature. Prepared tissues were mounted in Vectashield mounting media (Vector Laboratories, Burlingame, CA). Antibodies used were rat anti-DE-cadherin (1:20), mouse anti-Discs large (1:50), mouse anti-Matrix Metalloproteinase 1 (MMP1) (1:20), rabbit anti Activated Caspase 3 (AC3) (all from the Developmental Studies Hybridoma Bank at the University of Iowa), rabbit anti-b-galactosidase (1:2000, ICN/Cappel), guinea pig anti-Scrib (1:500, from D. Bilder, University of California, Berkeley). Secondary antibodies were Alexa 488 and 568 (Invitrogen) and Cy5 (Jackson ImmunoResearch). Immunofluorescence was analyzed on a Zeiss LSM700 confocal microscope using a 10X Neofluor (NA 0.3) air lens, 20X Apochromat (NA 0.8) air lens, or 63X Apochromat oil immersion lens (NA 1.4). Image J64 (National Institutes of Health, Bethesda, MD) was used to adjust brightness and contrast of whole images.

### Quantification of Proliferation in Wing Disks and Statistics

The degree of proliferation was quantified as the ratio of area of the GFP-positive area to total wing disk area to compensate for any effects that led to asynchrony in wing disc development. Total wing areas were determined by applying a Canny edge-detection algorithm to E-cadherin immunofluorescence images and filling in the resulting outlines. GFP-positive region areas were determined by applying a manually determined uniform brightness threshold to endogenous GFP fluorescence images. The proliferation index was computed as a ratio of GFP-positive area to total wing disc area. Image analysis and calculations were performed with a custom Matlab program, and code is available upon request to the corresponding author. P values were calculated via unpaired, two-sided Student’s t test. *p < .05, **p < .01, ***p < .001.

## Results

### Proliferation following disruption of the junctional versus basolateral polarity complex has different Mitogen Activated Protein Kinase (MAPK) requirement

Disruption of both junctional or basolateral polarity complex in *D*. *melanogaster* imaginal disc epithelium, a proliferating epithelium, can result in JNK-dependent apoptosis and compensatory hyperproliferation when apoptosis execution is inhibited [5,10,13]. To dissect and contrast the contribution of various signaling pathways downstream of junctional or basolateral polarity disruption that contribute to hyperproliferation, we set up a system in the *D*. *melanogaster* wing imaginal disc epithelium to quantify the extent of proliferation that occurred following disruption of either ABP polarity complex in the presence of the apoptosis inhibitor P35. The fly wing imaginal disc is a simple, two-layered, proliferating epithelium with an intact basement membrane. The wing disc undergoes a sequence of morphogenetic steps throughout development and to form the adult wing [14]. The availability of powerful genetic tools in *D*. *melanogaster* makes this a very useful system to interrogate signaling pathways downstream of polarity disruption in an intact epithelium. In quantifying proliferation, we controlled for possible asynchrony in development caused by genetic manipulation that might affect imaginal disc proliferation by quantifying a ratio of the GFP-positive proliferative area to the entire wing disc area. We developed custom image analysis software that calculated a ratio of GFP-positive *patched* (*ptc*) area to total wing disc area and used this as a measure of proliferation (S1 Fig, see Materials and Methods for further detail).

**Fig 1:**
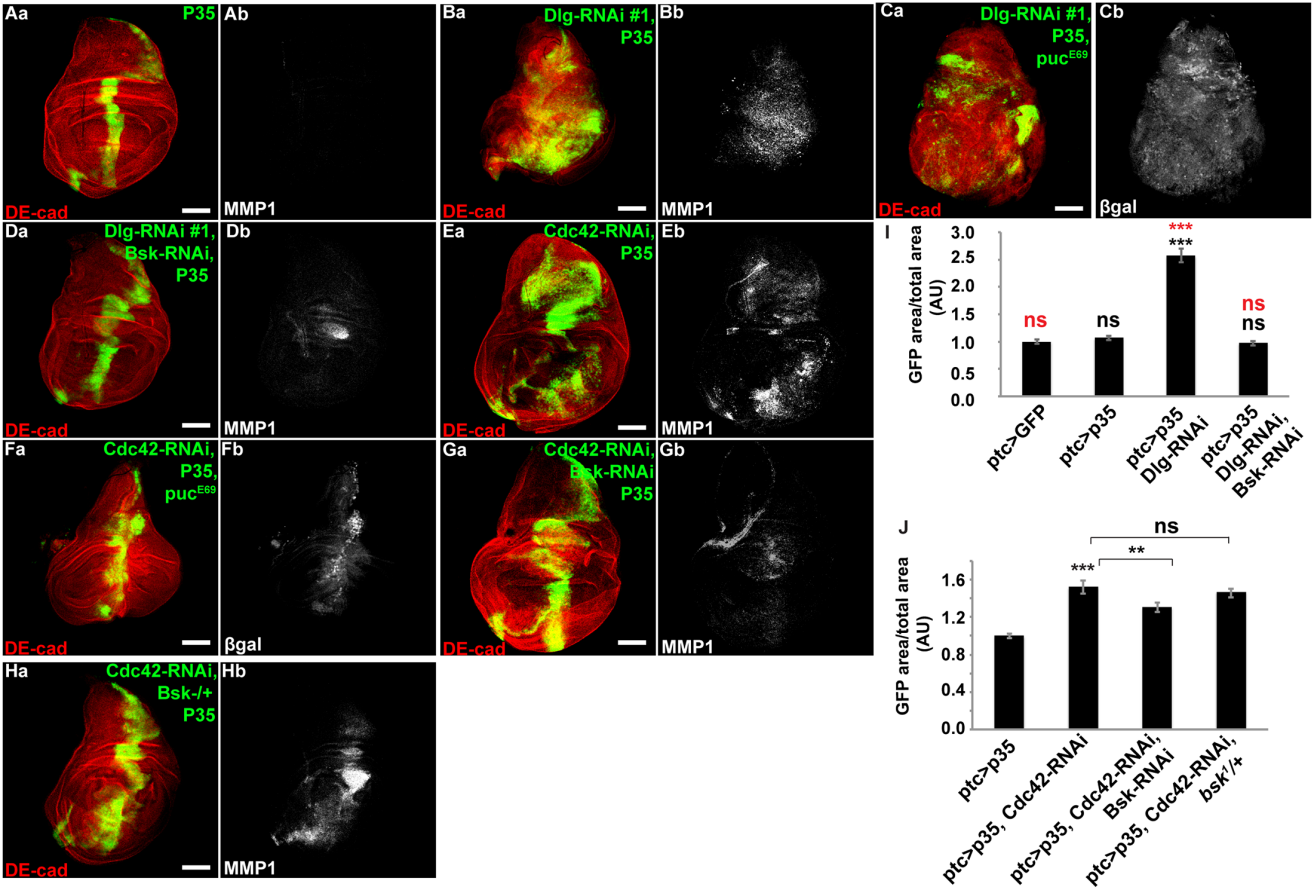
Junctional or basolateral polarity disruption in the presence of P35 leads to hyperproliferation, proliferation following basolateral complex disruption is dependent on JNK, proliferation following junctional complex disruption is largely JNK-independent. (A-H) Confocal immunofluorescent localization of DE-cadherin (DE-cad) (Aa-Ha), matrix metalloproteinase 1 (MMP1) (Ab-Bb, Db-Eb, Gb-Hb) and β-galactosidase (β-gal) (Cb and Fb) in larval wing discs expressing GFP with P35 alone (Aa and Ab), Dlg-RNAi and P35 (Ba and Bb), Dlg-RNAi and P35 in a heterozygous background of *pucE69* (*puc-LacZ*) (Ca and Cb), Dlg-RNAi, P35, and Bsk-RNAi (Da and Db), Cdc42-RNAi and P35 (Ea and Eb), Cdc42-RNAi and P35 in a *pucE69* heterozygous background (Fa and Fb), Cdc42-RNAi, P35, and Bsk-RNAi (Ga and Gb), Cdc42-RNAi and P35 in a *bsk1* heterozygous background, via *ptc-GAL4*. Quantification of GFP area/total wing disc area in tissues from conditions listed (I and J), n>14. Scale bars represent 100μm. Black statistical marks represent comparisons to the ptc>GFP control case, red statistical marks represent comparisons to the ptc>p35 case, unless otherwise indicated. AU—Arbitrary Units.

We first asked whether there was a difference in the requirement for JNK. The basolateral ABP complex was disrupted by RNAi-mediated depletion of Dlg in wing imaginal disc with *patched-GAL4* (*ptc-GAL4*, S2A Fig) in the presence of the apoptosis inhibitor P35. This resulted in imaginal disc hyperproliferation and tissue disorganization (Fig 1A and 1B, quantified in Fig 1I). In regions of hyperproliferation we observed increased activity of JNK, as determined by JNK-regulated MMP1 expression (Fig 1A and 1B) and the JNK reporter gene *puckered-LacZ* (puc^E69^) expression (Fig 1C). JNK activity was found to be critical for hyperproliferation, as when JNK was depleted with *Bsk-RNAi*, the hyperproliferative response was completely blocked (Fig 1D, quantified in Fig 1I). Since we saw a complete rescue of the hyperproliferative phenotype, we verified that Dlg protein level was indeed reduced by Dlg-RNAi when combined with UAS-Bsk-RNAi, UAS-P35, and UAS-GFP (S2C Fig), demonstrating that rescue was not merely a result of exhaustion of available GAL4 by multiple UAS promoters.

In contrast, when the junctional ABP complex was disrupted by Cdc42-RNAi in the presence of P35, depletion of JNK (Bsk-RNAi) or removal of a genomic copy of Bsk only partially rescued the proliferative phenotype (Fig 1E–1H, quantified in Fig 1J). Bsk (JNK) was activated following junctional ABP complex disruption, in the case of either Cdc42-RNAi, aPKC-RNAi or Baz-RNAi plus P35 (Fig 1E and 1F, S2F and S2G Fig)). These data indicated that the requirement for JNK in the proliferative phenotype resulting from ABP disruption differed depending upon whether junctional or basolateral polarity complexes were disrupted. Proliferation following disruption of the basolateral complex was largely, if not completely, dependent on JNK signaling, while proliferation following disruption of the junctional complex likely involves other downstream signaling molecules in addition to JNK.

### The MAPKKK, MEKK1, is uniquely required for proliferation following disruption of the junctional polarity complex

**Fig 2:**
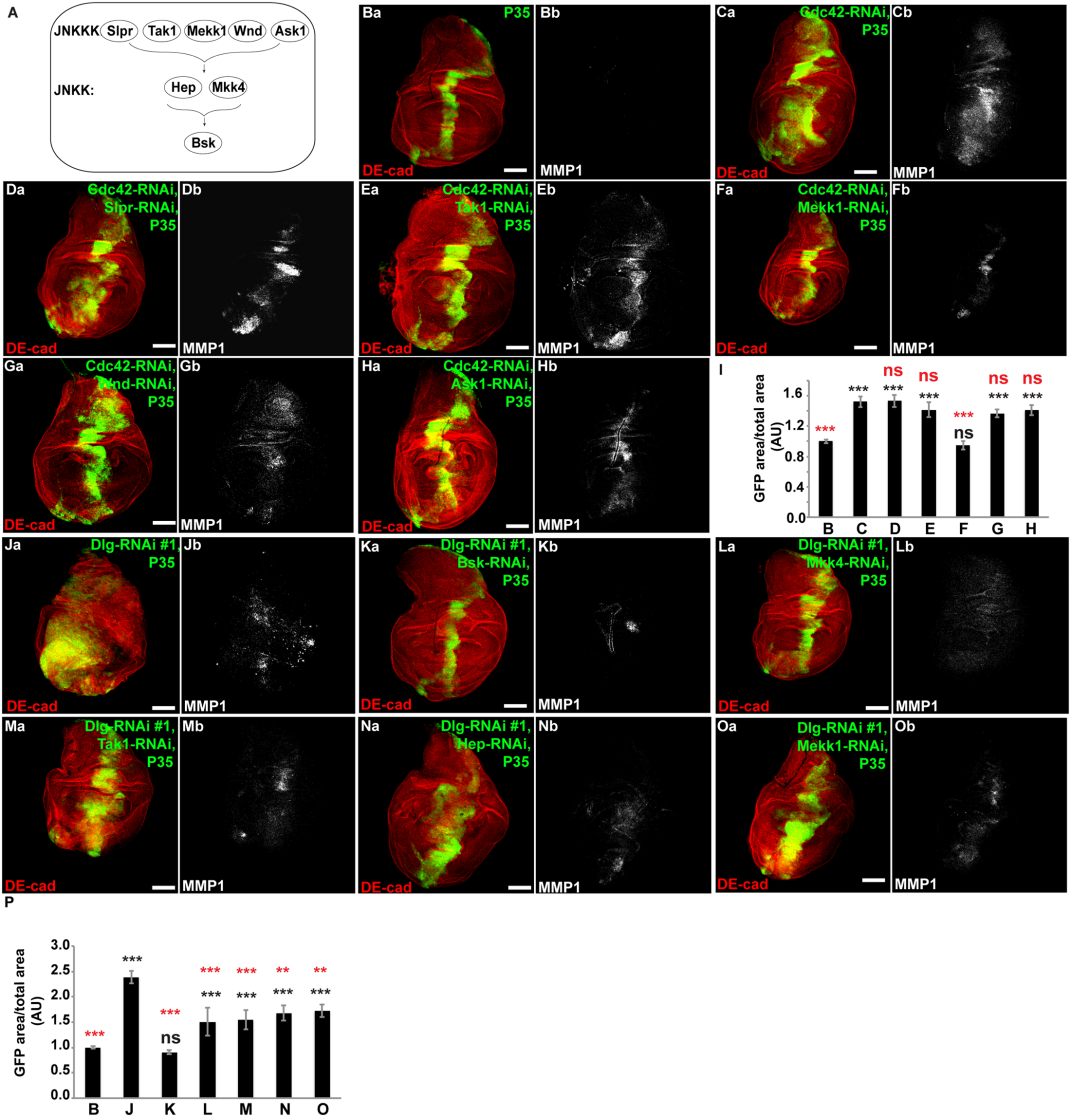
Hyperproliferation following junctional complex disruption specifically requires the upstream JNK-KK, Mekk1; hyperproliferation following basolateral complex disruption is dependent on multiple upstream kinases. (A) Schematic of JNK MAPK signaling in *Drosophila melanogaster*. (B-H, J-O) Confocal immunofluorescent localization of DE-cadherin, GFP, and MMP1 in wing discs expressing P35 and GFP alone (Aa and Ab), Cdc42-RNAi, P35 (Ca and Cb), Cdc42-RNAi, Slpr-RNAi, and P35 (Da and Db), Cdc42-RNAi, Tak1-RNAi, and P35 (Ea and Eb), Cdc42-RNAi, Mekk1-RNAi, and P35 (Fa and Fb), Cdc42-RNAi, Wnd-RNAi, and P35 (Ga and Gb), Cdc42-RNAi, Ask1-RNAi, and P35 (Ha and Hb), via *ptc-GAL4*. (I) Quantification of GFP area/total wing disc area in conditions shown in B-H, n>13. (J-O) Confocal immunofluorescent localization of DE-cadherin and MMP1 in wing discs expressing Dlg-RNAi and P35 (Ja and Jb), Dlg-RNAi, Bsk-RNAi, and P35 (Ka and Kb), Dlg-RNAi, Mkk4-RNAi, and P35 (La and Lb), Dlg-RNAi, Tak1-RNAi, and P35 (Ma and Mb), Dlg-RNAi, Hep-RNAi, and P35 (Na and Nb), and Dlg-RNAi, Mekk1-RNAi, and P35 (Oa and Ob), via *ptc-GAL4*. (P) Quantification of GFP area/total wing disc area in conditions shown in B and J-O, n>13. Statistical comparisons to “B” are shown in black, while statistical comparisons to “J” are shown in red. Scale bars represent 100μm. AU—Arbitrary Units.

Since JNK depletion alone was not sufficient to rescue the hyperproliferative phenotype when the junctional polarity complex was disrupted, we tested known upstream JNK-Kinase-Kinases (JNK-KKs–Slpr, Tak1, Mekk1, Wnd, Ask1) (Fig 2A) for the ability to rescue the proliferative phenotype. Surprisingly, RNAi-depletion of Mekk1, but not the other JNK-KK’s (Slpr, Tak1, Wnd, Ask), completely rescued the proliferative phenotype following junctional polarity complex disruption + P35 (Fig 2B–2H, quantified in Fig 2I). In contrast, the proliferative response following basolateral polarity complex disruption (*Dlg-RNAi*) + P35 did not require any single JNK-KK. Rather, Tak1, Hep, Mkk4, and Mekk1 all partially rescued the proliferative phenotype (Fig 2J–2O, quantified in Fig 2P), and none alone completely rescued the hyperproliferative phenotype like depletion of JNK (Fig 1).

### P38 is required for proliferation following disruption of the junctional, but not basolateral polarity complex

**Fig 3:**
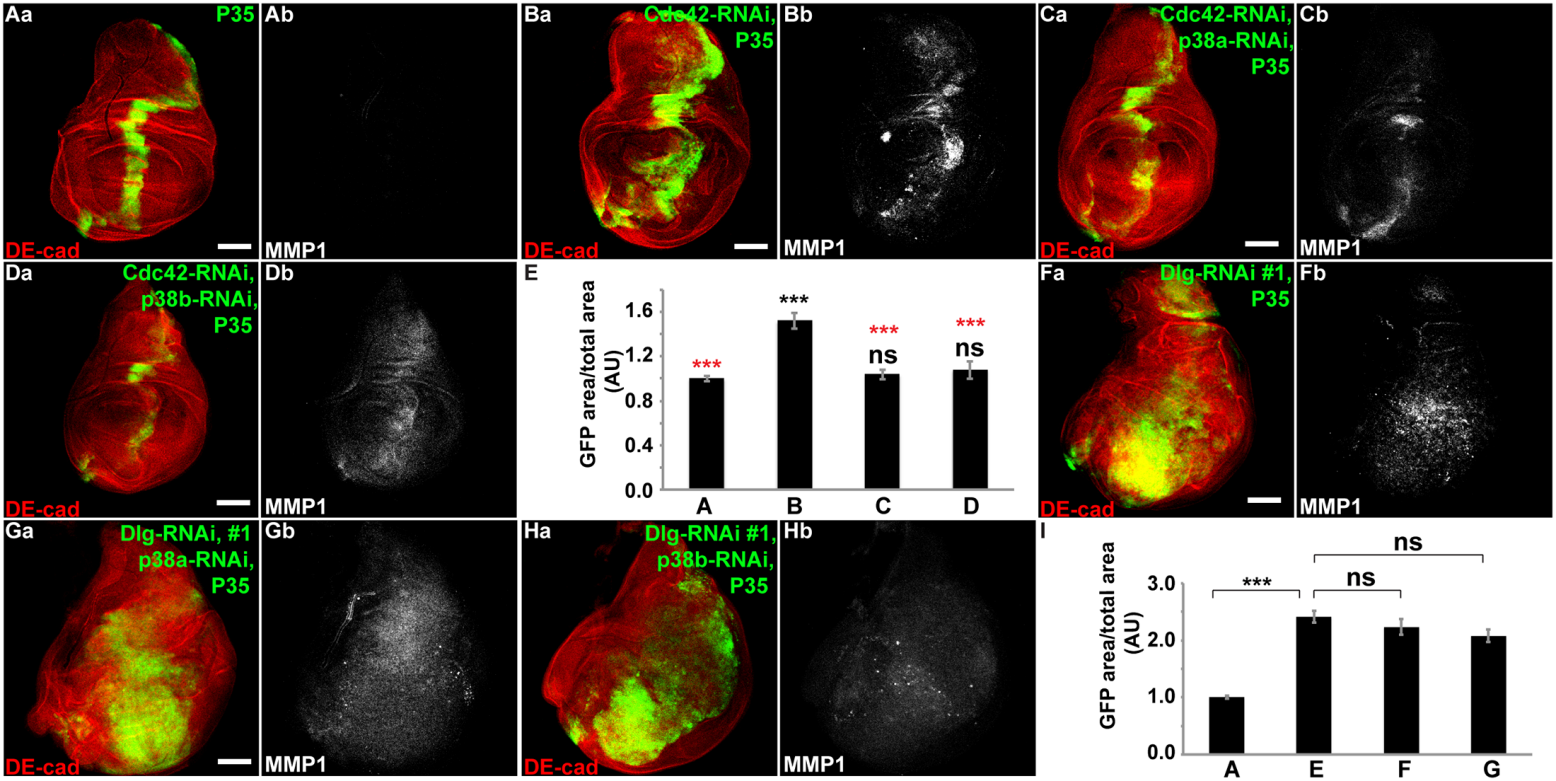
P38 MAPK is required for proliferation following junctional complex disruption, but not for proliferation following basolateral complex disruption. (A-D, F-H) Confocal immunofluorescent localization of DE-cadherin and GFP (Aa-Da), and MMP1 (Ab-Db) in wing discs expressing GFP with P35 only (Aa and Ab), Cdc42-RNAi and P35 (Ba and Bb), Cdc42-RNAi, p38a-RNAi, and P35 (Ca and Cb), Cdc42-RNAi, p38b-RNAi, and P35 (Da and Db), via *ptc-GAL4*. (E) Quantification of GFP area/total wing disc area in conditions shown in A-D, n>13. Statistical comparisons to “A” are shown in black, while statistical comparisons to “B” are shown in red. Confocal immunofluorescent localization of DE-cadherin and GFP (Fa-Ha), and MMP1 (Fb-Hb) in wing discs expressing GFP with Dlg-RNAi#1 and P35 (Fa and Fb), Dlg-RNAi#1, P35, and p38a-RNAi (Ga and Gb), Dlg-RNAi#1, P35, and p38b-RNAi (Ha and Hb), via *ptc-GAL4*. (I) Quantification of GFP area/total wing disc area in conditions shown in A and F-H, n>8. Scale bars represent 100μm. AU—Arbitrary Units.

Since Mekk1 depletion uniquely rescued proliferation following junctional polarity complex disruption much more significantly than depletion of JNK (*Bsk*), we considered the possibility that Mekk1 may be activating other downstream MAPKs, in addition to JNK (*Bsk*), to promote proliferation. Mekk1 can act as an upstream activator of JNK or p38-MAPKinase [15,16]. Thus, we tested whether p38 MAPK might be required downstream of junctional polarity complex disruption in proliferation regulation. Depletion of p38a or p38b via RNAi individually in Cdc42-RNAi + P35 wing imaginal discs resulted in a dramatic rescue of the proliferative phenotype (Fig 3A–3D), greater than depletion of just JNK (Fig 1). In contrast, depletion of p38a or p38b did not affect the proliferation response resulting from basolateral polarity complex disruption + P35, (Fig 3F–3H, quantified in Fig 3I).

In sum, these results indicated that the MAPK pathways downstream of junctional and basolateral polarity complex disruption +P35 mediated proliferation are different. The Mekk1-p38 signaling is the major MAPK pathway contributing to the junctional polarity complex phenotype, while JNK activation through multiple upstream kinases is the major MAPK pathway contributing to the basolateral polarity complex phenotype.

### Non-autonomous TNF signaling is important for the proliferative response following disruption of both junctional and basolateral polarity complexes

TNF signaling has been implicated as having either tumor-suppressive or tumor-promoting activity depending on biological context [10,13]. Disruption of the basolateral polarity complex, in a clonal context, induces JNK-dependent apoptosis through endocytic activation of TNF signaling, and when endocytosis in Dlg/Scrib-null clones was blocked, hyperproliferation of the epithelia ensues [10] Whether TNF signaling is important for the hyperproliferative phenotype observed following disruption of the junctional polarity complex has not been determined.

**Fig 4:**
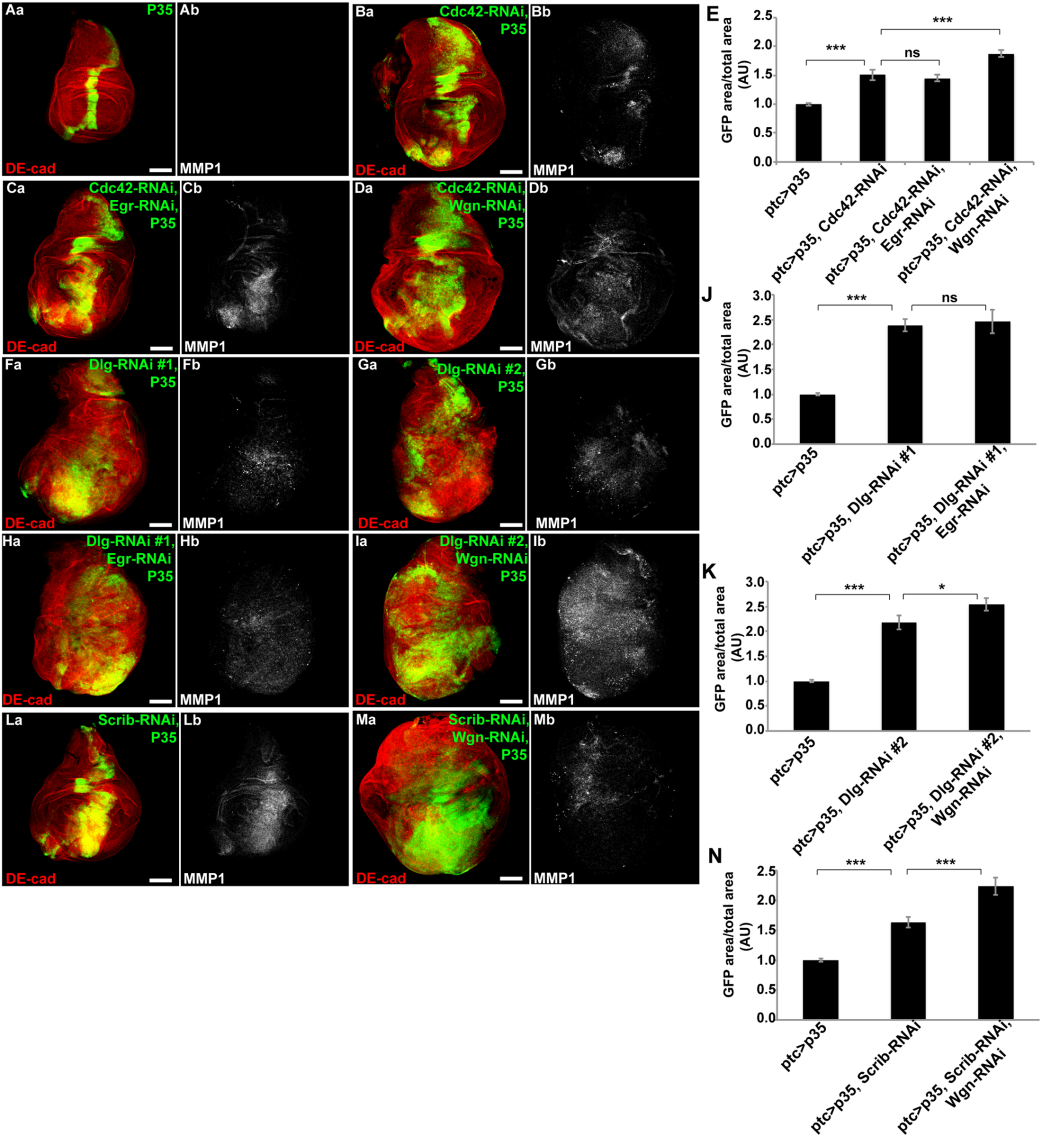
Non-autonomous TNF suppresses proliferation that results from junctional or basolateral complex disruption. Confocal immunofluorescent localization of DE-cadherin and GFP (Aa-Da), and MMP1 (Ab-Db) in wing discs expressing GFP with P35 only (Aa and Ab), Cdc42-RNAi and P35 (Ba and Bb), Cdc42-RNAi, Egr-RNAi, and P35 (Ca and Cb), Cdc42-RNAi, Wgn-RNAi, and P35 (Da and Db), via *ptc-GAL4*. (E) Quantification of GFP area/total wing disc area in conditions shown in A-D, n>26. Confocal immunofluorescent localization of DE-cadherin and GFP (Fa-Ia), and MMP1 (Fb-Ib) in wing discs expressing GFP with Dlg-RNAi#1 and P35 (Fa and Fb), Dlg-RNAi#2 and P35 (Ga and Gb), Dlg-RNAi#1, P35, and Egr-RNAi (Ha and Hb), and Dlg-RNAi#2, P35, and Wgn-RNAi (Ia and Ib), via *ptc-GAL4*. (J) Quantification of GFP area/total wing disc area in conditions shown in A, F, and H, n>18. (K) Quantification of GFP area/total wing disc area in conditions shown in A, G, and I, n>17. Confocal immunofluorescent localization of DE-cadherin, GFP (La and Ma) and MMP1 (Lb, Mb) in wing discs expressing Scrib-RNAi and P35 (La and Lb), and Scrib-RNAi, P35, and Wgn-RNAi (Ma and Mb), via *ptc-GAL4*. (N) Quantification of GFP area/total wing disc area in conditions shown in A, L, and M, n>18. Scale bars represent 100μm. AU—Arbitrary Units.

To assess whether autocrine TNF signaling played a role in the proliferative response following junctional polarity complex disruption, we co-expressed Eiger-RNAi (Egr, *D*. *melanogaster* TNF (verified in S3 Fig) with Cd42-RNAi and P35 via *ptc<GAL4*, *UAS-GFP* (Fig 4C). Depletion of Egr in cells expressing Cdc42-RNAi and P35 had no effect on the extent of proliferation (Fig 4A–4C, quantified in Fig 4E). However, depletion of the *D*. *melanogaster* TNF Receptor, Wengen (Wgn) resulted in increased proliferation of imaginal discs in which Cdc42-RNAi + P35 were present (Fig 4A, 4B and 4D, quantified in Fig 4E). This indicated that non-autonomous TNF signaling suppressed the proliferative response to junctional polarity complex disruption in apoptosis inhibited imaginal discs. Similar results were obtained when depleting basolateral polarity complex components (Dlg-RNAi or Scrib-RNAi) in combination with P35 (Fig 4F–4N). Thus, non-autonomous TNF signaling suppressed the proliferative response in epithelia when ABP was disrupted by either junctional or basolateral polarity complex disruption.

### Rho1/Rok/Myosin play opposite roles in regulation of proliferation following disruption of the basolateral versus junctional polarity complex

**Fig 5:**
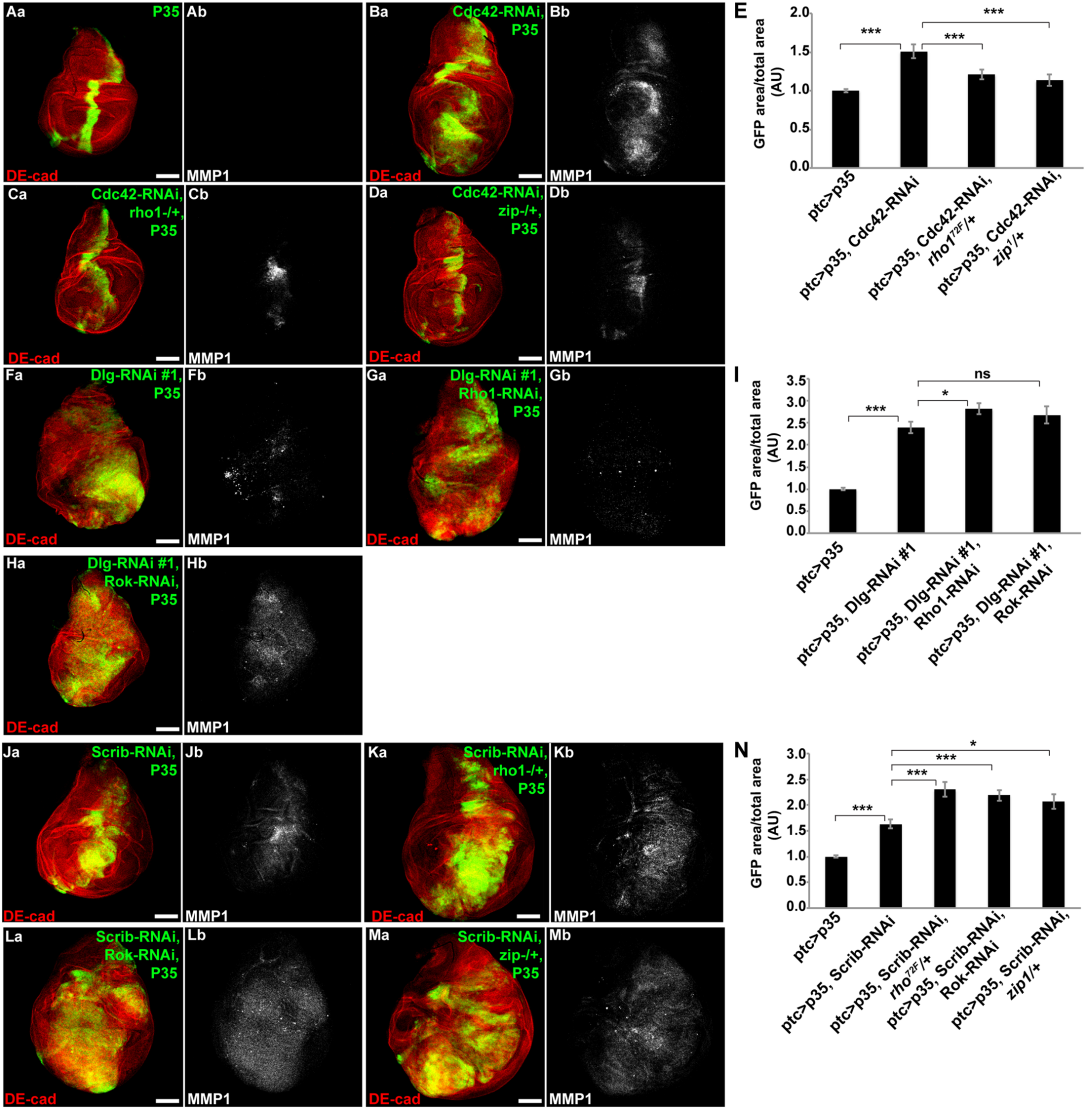
Depletion of Rho/Rok/Myosin suppresses proliferation following junctional complex disruption + P35, and promotes proliferation following basolateral complex disruption + P35. Confocal immunofluorescent localization of DE-cadherin, GFP, and MMP1 in wing discs expressing GFP with P35 alone (Aa and Ab), Cdc42-RNAi and P35 (Ba and Bb), Cdc42-RNAi and P35 in a *rho1*^*72F*^ heterozygous background (Ca and Cb), and Cdc42-RNAi and P35 in a *zip*^*1*^ heterozygous background (Da and Db), via *ptc-GAL4*. (E) Quantification of GFP area/total wing disc area in conditions shown in A-D, n>9. Confocal immunofluorescent localization of DE-cadherin, GFP and MMP1 in wing discs expressing GFP with Dlg-RNAi#1 and P35 (Fa and Fb), Dlg-RNAi#1, Rho1-RNAi, and P35 (Ga and Gb), Dlg-RNAi#1, Rok-RNAi, and P35 (Ha and Hb), via *ptc-GAL4*. (I) Quantification of GFP area/total wing disc area in conditions shown in A, F-H, n>17. Confocal immunofluorescent localization of DE-cadherin, GFP and MMP1 in wing discs expressing GFP with Scrib-RNAi and P35 (Ja and Jb), Scrib-RNAi and P35 in a *rho1*^*72F*^ heterozygous background (Ka and Kb), Scrib-RNAi, Rok-RNAi, and P35 (La and Lb), and Scrib-RNAi and P35 in a *zip*^*1*^ heterozygous background (Ma and Mb), via *ptc-GAL4*. (N) Quantification of GFP area/total wing disc area in conditions shown in A, J-M, n>18. Scale bars represent 100μm. AU—Arbitrary Units.

Previous work has demonstrated that disruption of the junctional polarity complex can increase Rho1/Rok/Myosin activity which then contributes to JNK activation and the hyperproliferation response [5]. Consistent with these results, we observed decreased proliferation in Cdc42-RNAi + P35 tissues when Rho1 or Myosin Heavy Chain (Zipper, Zip in *D*. *melanogaster*) levels were reduced (Fig 5A–5D, S4 Fig, quantified in Fig 5E). However, when Rho1, Rok, or Zip were depleted in Dlg-RNAi + P35 imaginal discs (i.e. basolateral polarity complex disruption), the proliferative response was actually enhanced (Fig 5F–5H and 5J–5M, quantified in Fig 5I and 5N). These results indicated that in contrast to junctional polarity complex disruption, the Rho1/Rok/Myosin signaling cassette acted as a brake against hyperproliferation following basolateral polarity complex disruption.

### The ERM protein, Moesin, regulates Rho-JNK signaling independent of the junctional polarity complex

**Fig 6:**
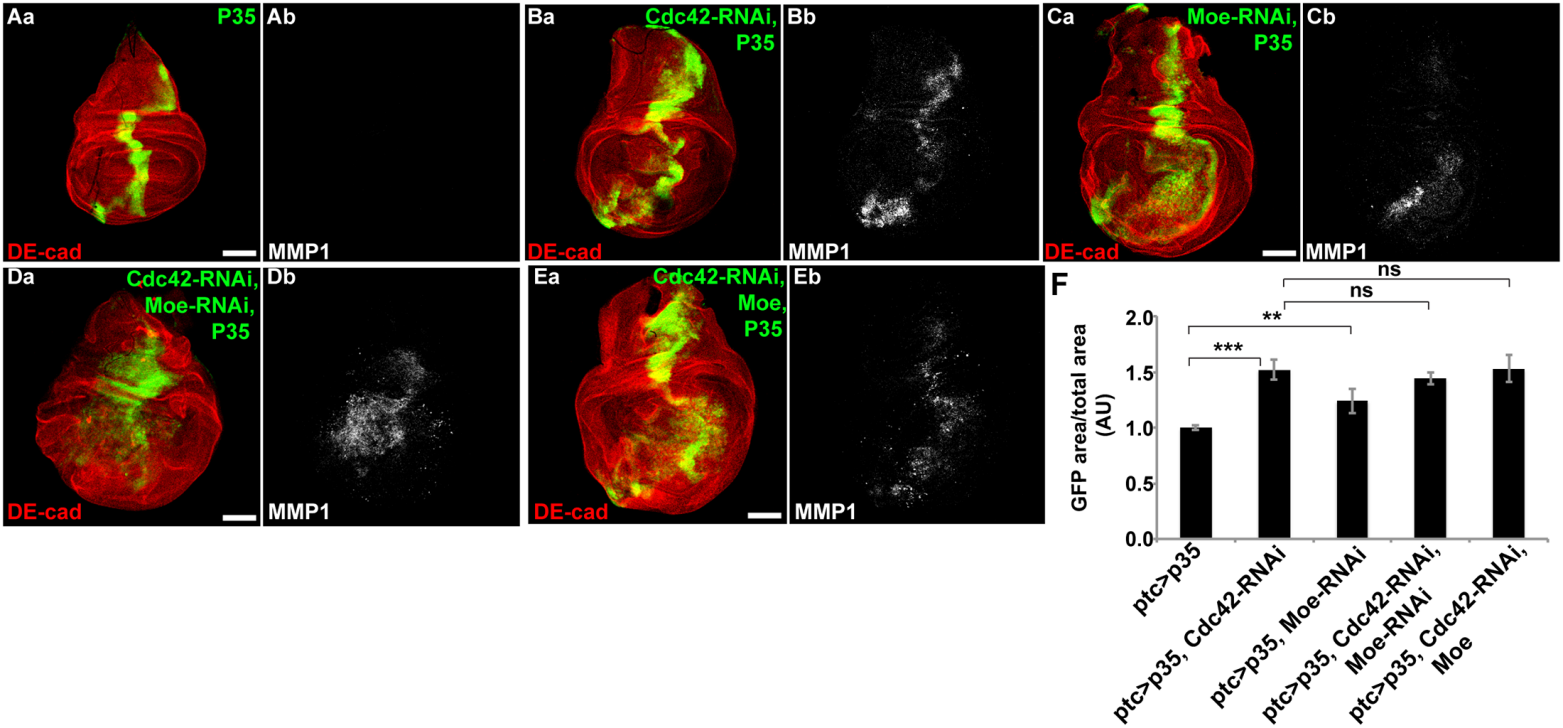
The ERM protein Moesin regulates Rho-JNK independent of the junctional polarity complex. Confocal immunofluorescent localization of DE-cadherin, GFP, and MMP1 in wing discs expressing GFP with P35 alone (Aa and Ab), Cdc42-RNAi and P35 (Ba and Bb), Moe-RNAi and P35 (Ca and Cb), Cdc42-RNAi, Moe-RNAi, and P35 (Da and Db), Cdc42-RNAi, Moe, and P35 (Ea and Eb), via *ptc-GAL4*. (F) Quantification of GFP area/total wing disc area in conditions shown in A-D, n>12. Scale bars represent 100μm. AU—Arbitrary Units.

The Ezrin/Radixin/Moesin (ERM) proteins are critical organizers of the cell cortex as they link cortical and transmembrane proteins to the actin cytoskeleton [17]. Moesin (Moe) the sole *D*. *melanogaster* ERM protein also negatively regulates JNK signaling and apoptosis through inhibition of Rho1 activity [18](S5 Fig). Since Cdc42 also serves as a negative regulator of apoptosis through Rho1 and JNK [5,11], we asked whether Cdc42 and Moe function in a shared pathway (e.g., Rho1) to inhibit the proliferative phenotype that results from Cdc42 depletion. If Moesin functioned in a linear pathway downstream of Cdc42 to negatively regulate Rho1 and JNK, then overexpressing Moesin might rescue the proliferative phenotype following Cdc42 depletion. Or alternatively, if Cdc42 functioned downstream of Moesin to negatively regulate Rho1, then overexpression of Cdc42 in the presence of Moe-RNAi might rescue the proliferative phenotype that results from Moe knockdown. To test this, we combined Cdc42-RNAi and P35 with either RNAi-depletion or overexpression of Moe. Cdc42 depletion or Moesin depletion individually combined with P35 both resulted in hyperproliferation and induction of MMP1 expression (JNK responsive gene) (Fig 6A–6C). Overexpression of Moesin did not prevent the proliferative phenotype in response to Cdc42 depletion, as would be expected if Moesin acted downstream of Cdc42 to restrict Rho1 activity (Fig 6A, 6B and 6E, quantified in Fig 6F). We were unable to assess how the combination of Moesin-RNAi with Cdc42 overexpression + P35 would affect proliferation as this resulted in lethality in pre-larval stages. Taken together, these results suggested that Cdc42 and Moesin did not function in a linear pathway to regulate JNK via Rho1.

## Discussion

Disruption of the ABP signaling network is known to contribute to increased proliferation and invasion of epithelial cells. These phenotypes have been observed in epithelia following disruption of either the junctional or basolateral polarity complex. However, the cellular signaling pathways activated to achieve this behavior is dependent on whether the junctional or basolateral complex is disrupted. Here we show that hyperproliferation following disruption of the basolateral complex was solely dependent on JNK MAPK, while disruption of the junctional complex caused proliferation that requires both JNK and p38 MAPK. Non-autonomous TNF signaling suppressed the proliferation that results from disruption of either complex. This could represent an epithelial-intrinsic or immunologic defense against the expansion of polarity-deficient cells in an epithelium. Our data suggested that this TNF-mediated suppression is indifferent to the genetic perturbation that led to the dysfunction. On the other hand, the Rho-Rok-Myosin contractility apparatus appeared to play opposite roles depending on which polarity complex was disrupted. This indicated that epithelial cells respond to polarity disruption via overlapping, yet distinct pathways depending on which components of the ABP signaling network are perturbed.

The fact that different MAPK signaling pathways are activated following disruption of the junctional versus basolateral polarity complex is, perhaps, not entirely surprising as these two complexes do serve unique functions. While the Par and Scrib complex have roles in regulating many shared processes, including intercellular junction homeostasis, cell polarity regulation, and RhoGTPase regulation, they are localized to different regions of the cell and therefore have unique suites of interacting proteins and signaling pathways.

Furthermore, during cell migration, apical-basal polarity proteins are reoriented to aid in establishing polarity along front-rear axis of migrating cells and play distinct roles in this process as well. Cdc42 is localized at the front of the cell and is important for protrusion formation in the direction of migration [19], and other members of the Par complex are important for spatiotemporal regulation of F-actin [20]. In immune cells, Par complex proteins localize predominantly to the leading edge, while Scribble complex proteins localize to the trailing edge where they regulate microtubule dynamics [21,22].

Our data demonstrated that disruption of ABP leads to JNK- or p38-dependent proliferation based on whether the junctional or basolateral complex is depleted. Why there is activation of one MAPK pathway versus the other in dysplastic polarity-deficient epithelial cells, and whether there are functional consequences, remains to be determined. This difference could result from distinct subcellular localization of JNK and p38 MAPKs, with JNK being localized more basolaterally and p38 more apically in the cell. An important function of the ABP network is to spatially segregate signaling networks such that cells can effectively integrate information from both the intracellular and extracellular environment, and transmit that information via appropriate signaling pathways [23–29]. The requirement of different MAPK’s downstream of ABP disruption could be an additional manifestation of this segregation. Apical activation of MAPK signaling by a Muc4-ErbB2 complex specifically leads to p38 activation, without activating JNK or ERK [30]. And there is much evidence linking basolateral polarity complex integrity specifically to JNK activity [1,10,13,31]. In plants, amplification of MAPK activity in distinct subcellular regions via interactions with polarity proteins is crucial for asymmetric cell division [32]. Thus, spatially restricted MAPKs could serve as local sensors that are activated as a result of the disruption of nearby polarity complexes, possibly following changes in local membrane composition, protein phosphorylation, or protein content in their respective subcellular domains.

Another contrast in the phenotypic response to junctional versus basolateral complex disruption was the opposite role played by the Rho/Rok/Myosin contractility apparatus. Disruption of the junctional complex leads to activation of JNK via Rho/Rok/Myosin, and depletion of any of these contractility components decreases the proliferative phenotype [5]. In contrast, depletion of Rho, Rok, or Myosin in the context of basolateral complex disruption dramatically exacerbated the proliferative phenotype. As apical-basal polarity and regulation of the actin cytoskeleton are closely linked, it is not entirely surprising that disruption of polarity activates downstream effectors via altered actomyosin dynamics. However, it remains unclear why opposite roles are played by the Rho-Rok-Myosin contractility apparatus following junctional versus basolateral polarity disruption. MAPK activity is responsive to actomyosin dynamics in both epithelial cells and myocytes [33–35]. Perhaps p38 activation in the apical region of the cell requires an increase in local actomyosin contractility, while JNK activation in the basolateral domain is somehow suppressed by actomyosin contractility. Alternatively, apical-basal polarity complexes could be differentially coupled mechanically responsive pathways, such as the Hippo pathway. Apical-basal polarity signaling, MAPKs, and the Hippo pathway have all been linked in the response to mechanical signals and disruption of cell architecture, such as with manipulation of actin polymerization [34,36–39]. The specific mechanisms underlying the opposite effect of Rho-Rok-Myosin depletion on proliferation following junctional versus basolateral polarity disruption remain unclear, and this is an important topic for future study.

## Supporting Information

S1 Fig Related to Figs 1–6. Wing Disk Image analysis. (A) Full wing disk showing immunofluorescence staining of E-cadherin (red) and expression of GFP via *ptc-GAL4* (green). (B) Identified regions: whole-wing (red outline) and Ptc region (green outline). (C) E-cadherin immunofluorescence with overlay of identified regions. (D) Ptc-GFP image with overlay of identified regions. Scale bars represent 100μm.
(TIF)

S2 Fig Related to Figs 1–5. Dlg-RNAi#1 and #2 effectively decrease Dlg protein levels, and Dlg-RNAi#1 is still effective when expressed in wing discs in combination with Bsk-RNAi and P35. Confocal immunofluorescent localization of DE-cadherin (DE-cad), GFP (Aa, Ba, Ca, Da, Ea, Fa, Ga, Ha), Dlg (Ab, Bb, Cb), MMP1 (Db, Fb, Gb, Hb), and Activated Caspase 3 (AC3) (Eb) in larval wing discs expressing Dlg-RNAi #1 (Aa and Ab), Dlg-RNAi #2 (Ba and Bb), or the combination of Dlg-RNAi#1, Bsk-RNAi, and P35 (Ca and Cb), Baz-IR and P35 (Fa and Fb), aPKC-IR and P35 (Ga and Gb), or Par6-IR and P35 (Ha and Hb) via *ptc-GAL4*, *or in MARCM clones of Dlg*^*1*^ *(Da and Db) or Cdc42*^*4*^ *(Ea and Eb)*. Scale bars represent 100μm in A-C and E. Scale bar represents 20μm in D.
(TIF)

S3 Fig Related to Figs 4 and 5. UAS-Egr induces ablated eye phenotype when expressed in developing *D*. *melanogaster* eye, UAS-Egr-RNAi, UAS-Wgn-RNAi, UAS-Hep-RNAi are effective at rescuing ablated eye phenotype towards wild type phenotype, Scrib-RNAi effectively reduces Scrib protein levels in developing *D*. *melanogaster* wing disc. Adult eyes expressing *gmr-GAL4* alone (A), or in combination with UAS-Egr (B), UAS-Egr and UAS-Egr-RNAi (C) UAS-Egr and UAS-Hep-RNAi (D), or UAS-Egr and UAS-Wgn-RNAi (E). Confocal immunofluorescent localization of DE-cadherin (DE-cad) (Fa) and Scrib (Fb) in larval wing disc expressing Scrib-RNAi, via *ptc-GAL4*. Scale bar represents 100μm.
(TIF)

S4 Fig Related to Fig 5. Rok-RNAi effectively reduces pospho-Myosin Light Chain staining in wing discs. Confocal immunofluorescent localization of DE-cadherin (DE-cad) and GFP (Aa), and phospho-Myosin Light Chain (pMLC) (Ab) in larval wing disc expressing Rok-RNAi via *ptc-GAL4*. Scale bar represents 100μm.
(TIF)

S5 Fig Related to Fig 6. Moesin-RNAi induces apoptosis, Moe-myc protein is expressed. Confocal immunofluorescent localization of DE-cadherin (DE-cad) and GFP (Aa and Ba), cleaved caspase 3 (AC3) (Ab), and myc (Bb) in larval wing discs expressing UAS-Moe-RNAi (Aa and Ab) or UAS-Moe-myc (Ba and Bb) via *ptc-GAL4*. Scale bars represent 100μm.
(TIF)
