# Supplementary figures and images for: Disruption of the Cdc42/Par6/aPKC or Dlg/Scrib/Lgl Polarity Complex Promotes Epithelial Proliferation via Overlapping Mechanisms
Source: PLoS One. 2016 Jul 25;11(7):e0159881. doi: 10.1371/journal.pone.0159881 (PMC4959776; doi:10.1371/journal.pone.0159881)

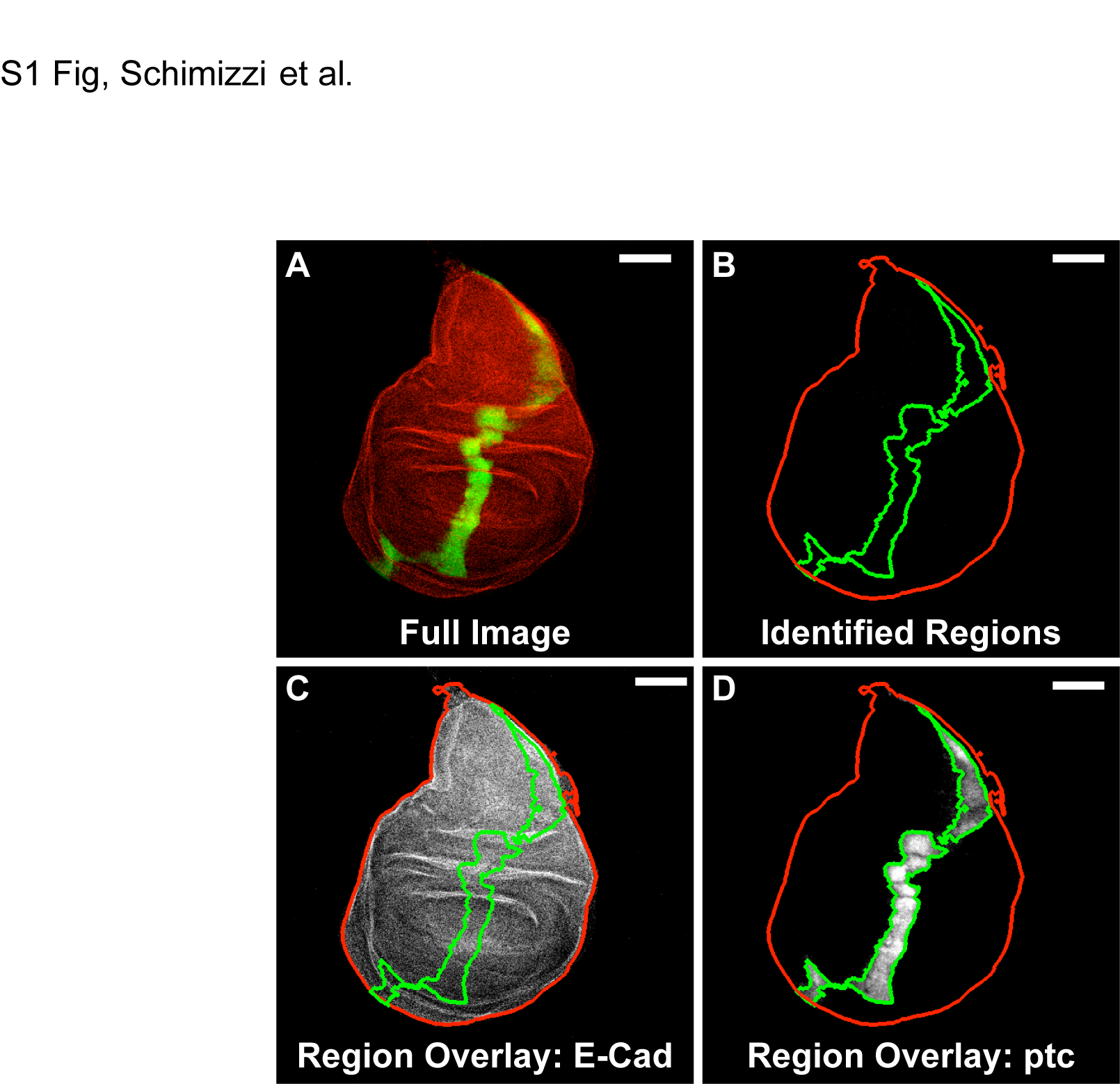

Supplement: S1 Fig — (A) Full wing disk showing immunofluorescence staining of E-cadherin (red) and expression of GFP via ptc-GAL4 (green). (B) Identified regions: whole-wing (red outline) and Ptc region (green outline). (C) E-cadherin immunofluorescence with overlay of identified regions. (D) Ptc-GFP image with overlay of identified regions. Scale bars represent 100μm. (TIF) [file pone.0159881.s001.tif]

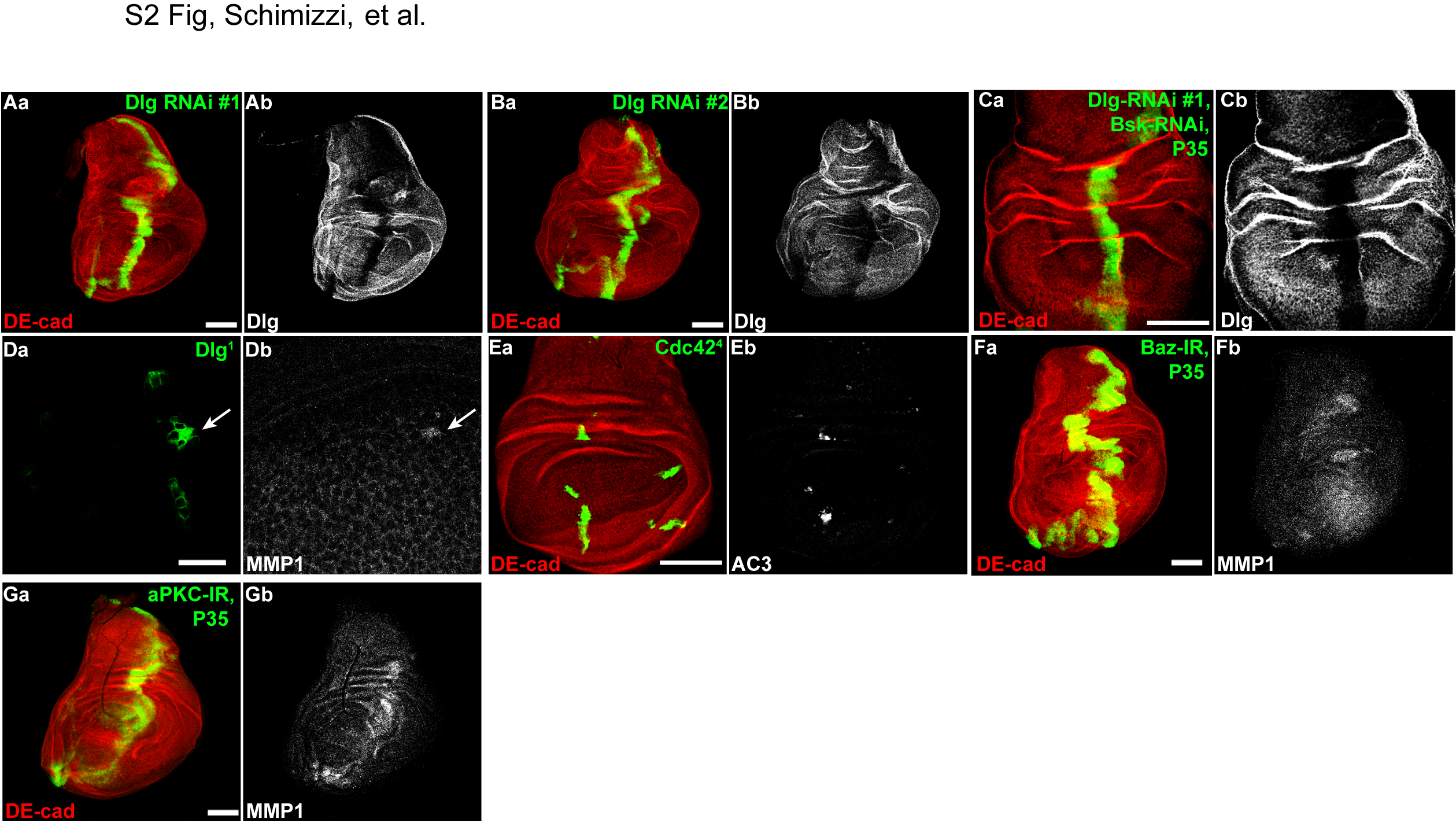

Supplement: S2 Fig — Confocal immunofluorescent localization of DE-cadherin (DE-cad), GFP (Aa, Ba, Ca, Da, Ea, Fa, Ga, Ha), Dlg (Ab, Bb, Cb), MMP1 (Db, Fb, Gb, Hb), and Activated Caspase 3 (AC3) (Eb) in larval wing discs expressing Dlg-RNAi #1 (Aa and Ab), Dlg-RNAi #2 (Ba and Bb), or the combination of Dlg-RNAi#1, Bsk-RNAi, and P35 (Ca and Cb), Baz-IR and P35 (Fa and Fb), aPKC-IR and P35 (Ga and Gb), or Par6-IR and P35 (Ha and Hb) via ptc-GAL4, or in MARCM clones of Dlg1 (Da and Db) or Cdc424 (Ea and Eb). Scale bars represent 100μm in A-C and E. Scale bar represents 20μm in D. (TIF) [file pone.0159881.s002.tif]

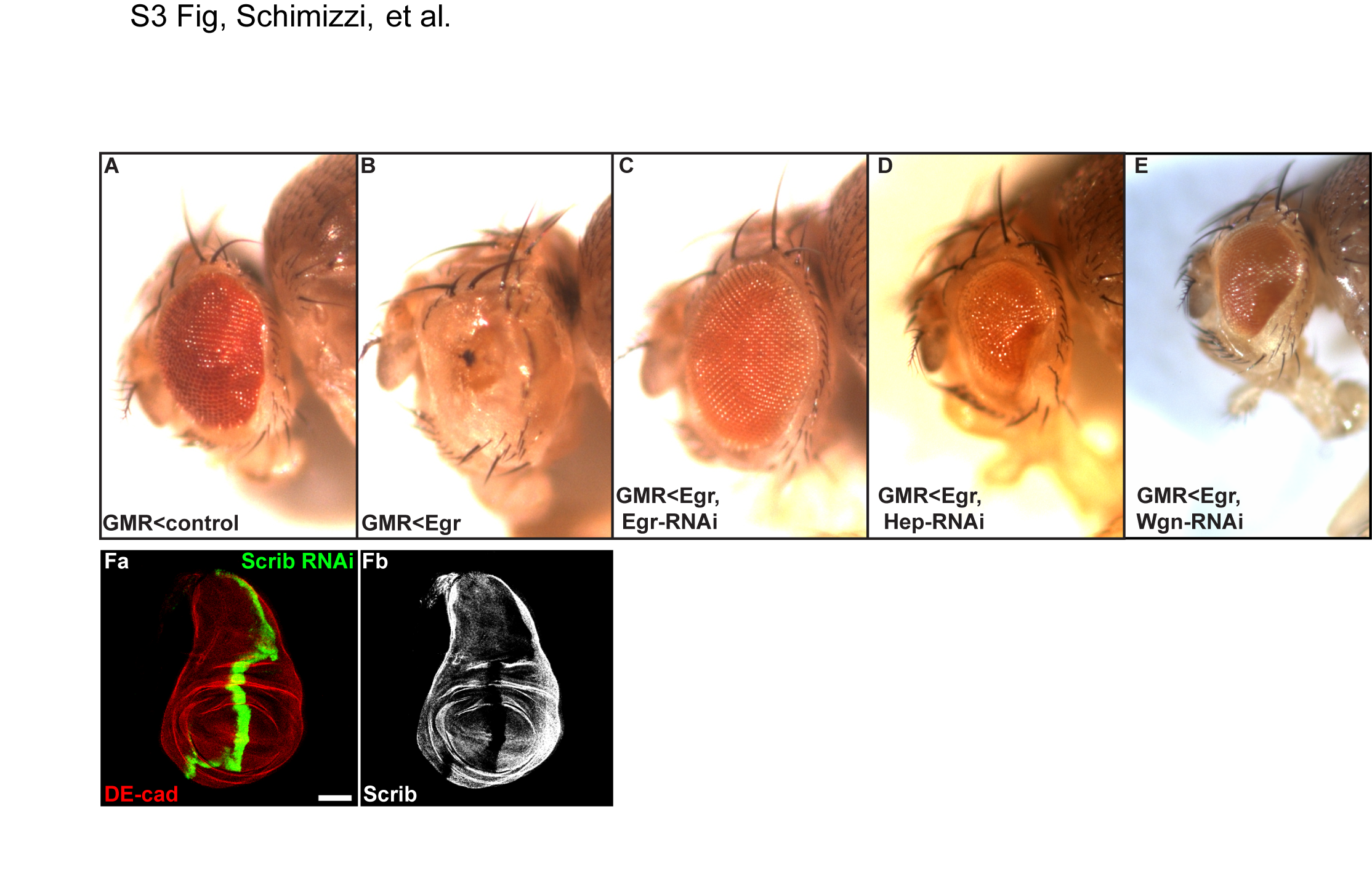

Supplement: S3 Fig — Adult eyes expressing gmr-GAL4 alone (A), or in combination with UAS-Egr (B), UAS-Egr and UAS-Egr-RNAi (C) UAS-Egr and UAS-Hep-RNAi (D), or UAS-Egr and UAS-Wgn-RNAi (E). Confocal immunofluorescent localization of DE-cadherin (DE-cad) (Fa) and Scrib (Fb) in larval wing disc expressing Scrib-RNAi, via ptc-GAL4. Scale bar represents 100μm. (TIF) [file pone.0159881.s003.tif]

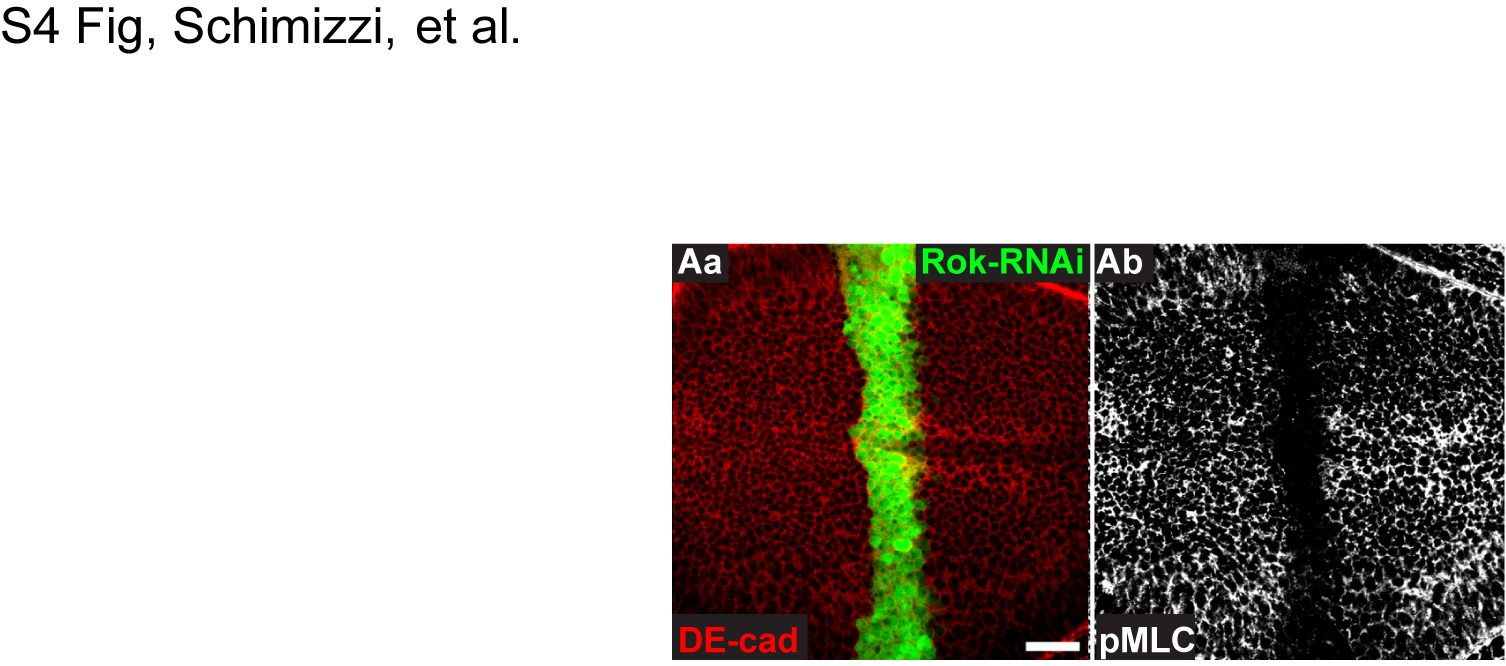

Supplement: S4 Fig — Confocal immunofluorescent localization of DE-cadherin (DE-cad) and GFP (Aa), and phospho-Myosin Light Chain (pMLC) (Ab) in larval wing disc expressing Rok-RNAi via ptc-GAL4. Scale bar represents 100μm. (TIF) [file pone.0159881.s004.tif]

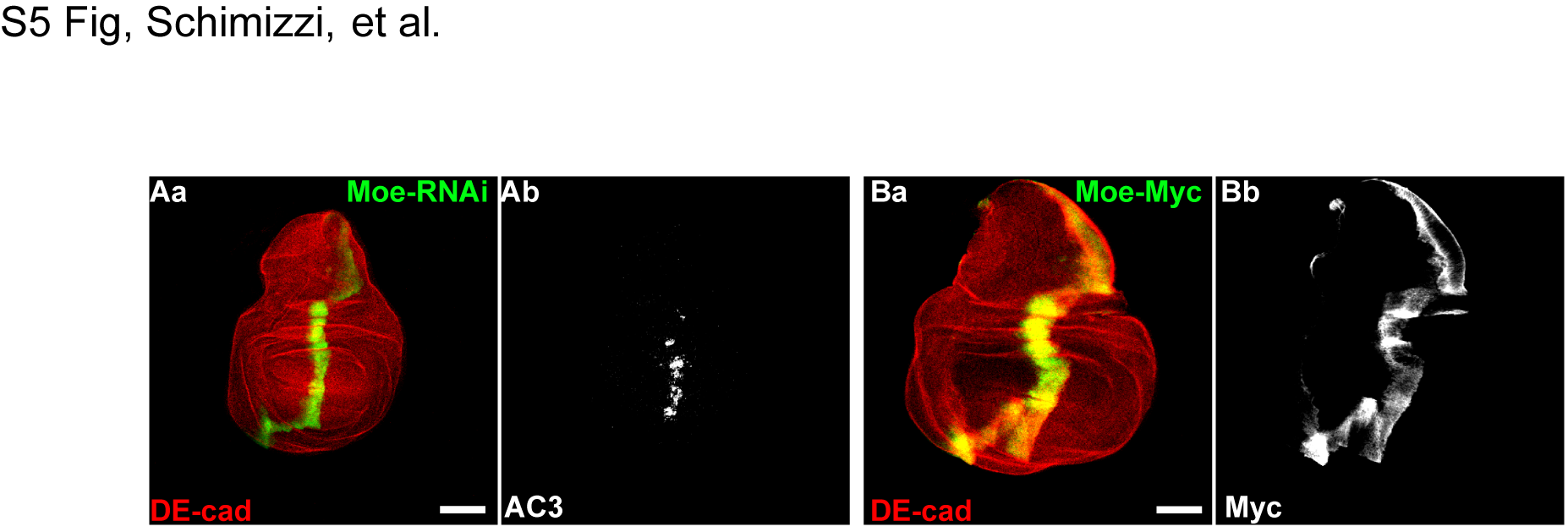

Supplement: S5 Fig — Confocal immunofluorescent localization of DE-cadherin (DE-cad) and GFP (Aa and Ba), cleaved caspase 3 (AC3) (Ab), and myc (Bb) in larval wing discs expressing UAS-Moe-RNAi (Aa and Ab) or UAS-Moe-myc (Ba and Bb) via ptc-GAL4. Scale bars represent 100μm. (TIF) [file pone.0159881.s005.tif]
